# Nervousness in the Male

**Published:** 1878-01

**Authors:** S. Weir Mitchell

**Affiliations:** Physician to the Philadelphia Infirmary for Nervous Diseases.


### ARTICLE IV.

### *Clinical Lecture on Nervousness in the Male.*

BY S. WEIR MITCHELL, M. D.,

Physician to the Philadelphia Infirmary for Nervous Diseases.

Many years ago, when we first began to sum up and classify the cases which came to my clinic, at the Infirmary for Diseases of the Nervous System, my assistants called my attention to the large number set down as general nervousness; and I then saw, with some surprise, that so very many of these were men. This was the more notable, because of course the persons who seek help at the Infirmary are mechanics and workingmen chiefly, and therefore hardly such as are presumed to suffer from nervousness.

Under more exact study, by means of referring the cases back to their causes, the number labelled general nervousness has been somewhat lessened, but in each year's report still appears a group in which either general nervousness is the dominant condition, or which at all events I find myself unable to classify under any other heading.

We rarely see this condition delineated in the books. You may read whole text books about nervous diseases, and see no mention of this striking, this annoying, this disabling condition; or, if spoken of at all, it is as if it were entirely the sad prerogative of woman. We may find it useful, therefore, to look back over some of our cases to see how they originate, what precisely are their symptoms, and to what extent and in what manner we have power to relieve them.

In so doing I do not desire to be considered as wishing to make of general nervousness a disease. Yet does it sometimes present itself here and elsewhere, with so little apparent relation to casual conditions, and with so distinct a group of clinical peculiarities that it is at times hard to do otherwise than so to treat it. For the most part it is the offspring of obvious defects in the nutritive supply, and is plainly related to coincident or precedent changes in the nervous or vascular systems. Sometimes too it is ephemeral, sometimes it is only too lasting, and in a few cases it is due to original defects, and arising at birth is carried to the grave.

As concerns many other symptoms, so also of this. It does not answer to accept the statement of the patient that he is nervous; we want also to know what he means by nervousness; and this brings me to the definition of the term. Perhaps a couple of illustrations may aid us in this study. The first I shall offer is a very good example of a somewhat rare cause of this state, and is a type of the complex symptom or disorder as perfect as we can desire.

C. J., æt. 28, about 5 feet 10 inches high, and weighing 135 lbs., tells me this story: At the age of eighteen he was well, active, and of rather cool and quiet temperament; not in the least a nervous lad. At this time he began to learn segar making, and has been at the trade until a year ago, when he gave it up. He has been in all ways temperate, and curiously enough has not learned to smoke or chew. This trade has, however, been, in my opinion, disastrous to his nervous system, and this is not an exceptional case, as I have seen others who had been in like manner injured by the same occupation. By slow degrees, but more and more each year, this young man has developed a nervous constitution, which he attributes to the work he did, a conclusion in which I fully agree. At last it drove him from the trade and now he is here for relief. Let us look at the range of symptoms he presents.

He is sallow, and inclines to anæmia. His appetite is fair, his digestion uncertain, good to day, bad to-morrow; his bowels much like his digestion. I can detect no renal or intestinal disease, and he has never had ague or syphilis. His circulation is poor; his heart feeble; it beats when he is lying down 85, and rises abruptly to 100 when he stands up. A sudden sound, a slight scare, an unlooked for visitor, gives him palpitation of the heart, sense of throbbing in the limbs, and coldness of hands and feet, with local sweats.

He has become emotional also, and gives way readily to grief, or to mirth. He sleeps ill, dreams much, and is liable like a child to night terrors. As to exercise he can work as well as ever, but thinks he tires more than he should do; and he adds finally, I used to be brave as any body, but now I am afraid of everything. Since he gave up segar making he has been slowly gaining ground, under the use of a variety of tonics.

As a second illustration I turn to my note-books kept during our late civil war.

C. J., æt. 30, a large, vigorous man, was one of the leaders of the gallant storming party which took Fort Fisher. He had, before this, led a life of adventure and danger. Probably it would have been hard to find a more cool, quiet resolute soldier. He was shot through the right lung, and the ball emerging, entered the arm, and injured the median nerve. He had a long series of lung troubles, and the terrible anguish of causalgia (burning pain) in the hand, exhausting suppuration, and constant pain, reduced him in three months to a slightly covered skeleton, and transformed him morally into a peevish, timid child. In this condition he was a type of a nervous man. A sudden noise, an unlooked for letter, the morning visit of the doctor, set his heart beating and made his limbs cold. At the prospect of a hypodermic injection he would shed tears, and he became annoyingly susceptible to the irritative influence of sounds and light.

When we come to ask in what general nervousness consists, we shall find, I think, that its prominent peculiarity lies in over-excitability. This is not all of it, but it is a large, perhaps the largest part. The patient is too easily moved, too readily excited. The strong man becomes like the average woman, the woman like the unschooled child, and the symptoms for the most part are not utterly abnormal, but are exaggerations of normal conditions. Many of the characteristics exhibited by the nervous man on the slightest provocation are seen in the normal man at times on rare occasions, under exposure to unusual emotion. A nervous man receives a telegram; he becomes pale, his heart throbs, and he opens the envelope with a trembling hand. It proves to be a message of no moment, but the effects last for an hour. A healthy man might have shown like disturbance under the influence of some sudden announcement of the gravest calamity, while we shall meet with men who under no circumstances show the least outward sign of emotion while in health. The mass of men are naturally free from nervousness, and perhaps the hardening education which the man undergoes is a means of forcing him to repress emotion and to restrain the exhibition of feeling until restraint develops habit. On the other hand, the lonelier lives led by women leave them more free in this respect, and all their training lies too much in the opposite direction to that of men. In either sex early education may do much to restrain acquired or inherited nervousness, while it is quite sure, as I have many times seen, that the most hardy children may be made nervous and emotional by over-indulgence, and by allowing them to grow up without having learned this life's most urgent lesson, that of self-restraint. I have said that the condition I call general nervousness is mainly characterized by a certain readiness to be excited, and it remains to point out the rest of the clinical peculiarities which go to make up a picture which it is perhaps difficult to make sharply definite. With the tendency to be easily moved there is also a certain apprehensiveness which applies both to the possible effects of outside influences, and to the symptoms of the patient himself. What he feels he exaggerates, and he is alarmed by what he feels; knowing also how certain agencies affect him, he dreads them, and thus learns to shun his fellows and so to avoid the incidents which contact with men brings about. Very nervous persons are also apt to suffer from a sense of confusion upon sudden or prolonged use of the mind, and from being in a crowd, or where there are many speakers.

In all such men, anxiety fear, embarrassment, are prone to occasion some disturbances in the sphere of motor activities, which are, to begin with, never, in such cases, quite what they should be. Thus in acquired nervousness, the hand is apt to become tremulous, and under emotion of any kind this may increase, so that, when conscious of being observed, it beomes impossible even to sign the name. Under more violent emotion greater disturbances may occur and the tremors become larger, and even pass into the degree of spasm, local or general. All painful emotion is competent more or less to disturb and enfeeble the normal capacity for movement, and in nervous people emotion becomes doubly capable of weakening the limbs, of affecting the speech, even to inhibition of vocal utterance, and of showing itself in the play of the features.

In some cases, the mental or emotional impression disturbs the vaso-motor system, and we have then spasm or dilatation of vessels, violent arterial throbbing, irregular and excited cardiac action. How the extreme of nervousness acts on the battle-field is well known. Sometimes like annoyance is occasioned in acquired nervousness, and emotion provokes urination or motion of the bowels. I have seen more than one nervous man who on leaving home in the morning would be turned back again and again to make water. Mr. Carpenter has long since called attention to the fact that when we watch our automatic movements, and try to make them voluntary, we at once make them difficult. As in nervous people too much attention is apt to be paid to the processes which go on in the body, we find in them in an exaggerated degree just such difficulties in the act of swallowing, of micturition, and, as I have said above, in those grouped habitual movements, such as writing, which have become almost automatic. Exaggerated states of nervousness give rise in men, as in women, to the motor disturbances, which are said to characterize hysteria, and even to many of the psychical peculiarities of that strange state, although only once have I seen a man exhibit the whole range of these phenomena.

The sleep of men in this condition is rarely good, nearly always they go to sleep with difficulty, dream unpleasant dreams, and wake often or early.

When we come to ask ourselves what is the state of the nerve structures which gives rise to the symptoms described, we are somewhat at a loss. It is easy in a large majority of cases to feel sure that it is, however caused, a state of feeble or tardy nutrition with which we have to deal, that it is in turn associated as usual with an extremely unstable state of circulation in the nerve centres, so that states of congestion at one time, and at another of anæmia from vasal spasms may prevail; but, if we seek beyond obvious clinical conclusions to determine just how the nerve cells are changed, we are forced to pause and admit that the theory of feeble nutritive supply fails to explain many of the cases of sudden nervousness such as those which arise from mental shock. I saw a curious case of this kind some years ago.

A young man, æt. 18, while attending his father's funeral, slipped on the wet ground and fell into the grave. From this time he became strangely nervous, was startled readily, grew timid and apprehensive, and at last became unable to write while any one was watching the act. The tremor thus caused, at last so increased that he had to give up a clerkship which he held. This condition of system came as it were in a moment, and converted a quiet somewhat resolute person into a nervous invalid. I might quote other cases, the one I have given may suffice. This man remained in good physical health, and was thus in contrast with most cases of acquired nervousness, which as I have already said, are at least associated with states of feebleness, if they be not always traceable to these, but although such is distinctly true, the form of trouble causing weakness, innutrition, and anæmia varies, and with its variation the intensity of the nervousness, and even its quality varies. Nor must we lose sight at any time of the character of the patient, for this also must make up a part of the case, and it will be one thing to deal with a case of congenital nervousness made worse by circumstance, and quite another to treat a person at one time firm and cool, and who has merely become nervous through disease or suffering.

It may repay us to run over the causes of nervousness, with such illustrations as our case books afford.

I have mentioned already one case as an illustration of the influence of enfeebling wounds. In like manner we find sometimes extreme cases of nervousness following fevers or exhausting diarrhœas. Every old army surgeon, who recalls the Chickahominy diarrhœas, will recall too the dreadful condition of nervousness to which it reduced many persons.

The connection of general nervousness with neural maladies is also of interest. The victims of general sclerosis, and still more those of paralysis agitans, are apt to suffer horribly from acquired nervousness, while in posterior sclerosis (locomotor ataxy) the patients are often singularly free from nervousness, unless they continue to suffer from the atrocious pain which sometimes continues throughout the whole course of the disease. It is amazing to learn the extent of brain disease which may exist without making the sufferer excitable, timid, tremulous, or in a word nervous. So much may also be said of many cases of brain tumours, and of very many hemiplegias, while affections of the pons and of the sensory tracts, involving heminæsthesia with pain or obscure unilateral sensations of burning are sure to cause horrible nervousness.

How far visceral disease may cause it depends a good deal on the individual, but more on the organ attacked, as we well know. A poet has said "hope springs eternal in the human breast," and certainly, to judge from the psychical peculiarities of pulmonary disease, he was fully justified. On the other hand, the nervousness and depression which accompany disorders of the abdominal viscera are well known, and gave rise to the adage of the Presbyterian divine that "no man dies a triumphant death who dies of disease below the diaphragm."

The genito-urinary tract in man furnishes in disease remarkable illustrations of the extreme of general nervousness. Distressing examples are supplied by some of the many cases of seminal emissions which we see, and sometimes sexual excesses give us equally signal instances.

The worst cases I have seen were those of sexual excess in the young. I was consulted four years ago by a gentleman, aged 27, from Tennessee, who told me, that when a mere lad he cohabited with a girl several years his senior, and the intercourse was kept up without suspicion until the girl had become a teacher, and the lad was himself seventeen years old. At this time he ceased to see her, but the mischief was done. From the age of thirteen, he had become excitable, emotional, and tremulous on the least sudden noise or mental shock. This increased so, that when he left school at seventeen, he began to shun society owing to his want of self-control. He then improved during a year spent in the country, but the work and confinement of a counting house again brought back his old trouble, and he began, after two years, to lose the power to write owing to increasing tremor of the hands; a more distressing case I never saw.

Nervousness is a frequent accompaniment of that form of head trouble, about which there are so many theories, but which is most common in the young, or at the close of middle age. I presume that the term nervous exhaustion is as good a label as any. It does not always lead to great nervousness, but it does do so sometimes, as in this rather curious case, which I saw but lately.

M. L., æt. 58, the leader of the bar in a Western city, was forced some years ago to carry on the trial of a most important case, while his wife lay dangerously ill. From this time he began to find that he felt embarrassed when rising to speak, and that he was obliged to urinate always before speaking in court. The mere knowledge of these facts began to trouble him, and soon after he found that his emotions were less under control than they had been. At last one day when about to sign his name, he found that his hand shook, because two gentlemen who were to act as witnesses were watching him. From this time he could no longer write, when overlooked, unless he made the most earnest effort. With these disabling conditions, he began to fail in vigour and appetite, and to become excessively restless and irritable. Under fortifying treatment with long absence from home he became entirely well.

While epileptics are often quite free from nervousness, frequent attacks of vertigo, whether due to aural disease or to other causes, are almost certain to result in general nervousness of the most distressing kind. Where vertigo occasions the condition which I recently described (*Med. Reporter*, lecture on Vertigo) as the vertiginous status, this is notably the case. In this state frequent onsets of vertigo give rise to a permanent sense of instability, and also to confusion whenever prolonged or intense thought is attempted. This I venture to call mental vertigo.

Tobacco is of all causes the most potent in giving rise to general nervousness. Used in excess, it makes the heart irritable and feeble, produces tremor and insomnia, and may develop even in sturdy people a high type of nervousness. Its potency is singularly well seen in its effects on people by nature nervous, and in the remarkable manner in which it disturbs many ataxic patients, or those who have paralysis agitans.

I have now an ataxic case which well illustrates this peculiarity. The patient has ceased to have pain, and seems for years to have been at a stand still. He has made one of those curious pauses in the disease which flatter the patient with the hope of recovery, and delude too hopeful therapeutists. The patient is excessively fond of tobacco, and despite all that it costs him will now and then venture to try it again. The result is interesting and always the same. He becomes, after two segars, so emotional that noise, a telegram, a letter to be opened unnerve him, and his voice fails if he has to address a stranger. He acquires tremor of the hands, and writing becomes impossible for a day or more.

Here also is a strange example of neurotic states in the male caused by, first, a peculiar constitution, and, secondly, by extreme abuse of tobacco. Had it been a woman's case, no one would have failed to label it hysteria.

P. L. T., æt. 24, a young merchant, belonged to a family, which offered in the last generation two cases of melancholia and one of epilepsy. Mr. T. was the sole remaining representative. He was always a delicate looking man, of nervous temperament, quick, restless, and anxious. At the age of sixteen years he began to chew and to smoke, and used tobacco in both forms to excess—up to his twenty-third year, when I saw him first. I learned then that he had become more and more nervous, and had twice had fits of alternate tears and laughter, seemingly quite uncontrollable. The night I saw him, he had had a fall, from which he did not rise, but called for help, insisted that his thigh was broken, and was at last carried home on an extemporized litter. When I examined him, his right leg was rigid, the leg extended. The spasm lasted for some hours, while I sat and watched him. When it gave way and the legs began to twitch, he had a general convulsion, then a period of apparent insensibility, then a series of such fits, and at last a remarkable condition of general rigidity. The series ended with an enormous flow of thin pale urine.

These phenomena recurred at intervals for two years, and I have over and over witnessed in him nearly the whole range of hysterical symptoms. His last attack began with fits of crying, and ended in hemi-anæsthesia and partial loss of power on the same side.

Mr. L. was made well by giving up tobacco, and by a long course of careful physical training.

Among the most potent causes of general nervousness in man, as in woman, is a state of anæmia. It is probably the immediate cause in some of the cases credited to pain—to exhausting discharges, and to maladies of assimilation, to which I have already alluded, and at all events it is a condition never to be lost sight of in the treatment. A remarkable example is to be found in the case of Mr. L. P——, recorded at page 89 of my little book on *Fat and Blood*, second edition. In this instance the anæmia was remarkable, and the nervousness not less notable.

I have seen children reduced to a state of pitiable nervousness, with feebleness of will and convulsive states not epileptic, owing to unsuspected albuminuria. I am now attending such a case in a lad of the age of thirteen.

I am unwilling to end without some allusion to treatment, but as to this I must be brief.

A large part of the treatment of nervousness must of course, resolve itself into the treatment of anæmia, of defects of nutrition, of mal-assimilation, of dyspepsia. I have said that, in one way or another, poverty of blood is most often the true difficulty. It may be due in turn to this cause or to that, but when these have been cured, or helped, there will yet be left the anæmic state, and in some people, when this exists, it is strangely intractable. On this matter, however, I have nothing to add here to what I have recently said concerning the treatment of obstinate states of anæmia, either with or without loss of flesh. The slighter grades are, of course, to be met in the usual ways familiar to all physicians.

But nervousness, whatever be its parentage, demands something more than this. Only too often the symptoms, which make up this annoying state, continue after we have amended the blood losses, which undoubtedly occasioned them. There is, perhaps, in this fact but another example of the persistency of morbid habits. The man whose self-confidence has once been rudely shaken does not speedily reacquire hardihood in the face of disturbing impressions, and we have also to deal with the many instances of nervousness which arise out of moral causes or are of unknown birth. In the cases of women, we can often aid by secluding them for a time from the kind of troubles which keep up the habit; and with men we may reach the same end by cutting them off from their routine lives, through the help of travel and change of scene. Above all, it is useful to give to such persons the out-door life of field sports; and where these cannot be had, it is often wise to induce the sufferer to undergo some gradual course of physical training, since this has in many cases the happiest results. I have over and over again seen the most helplessly nervous men lose their annoyances under such a plan, but it requires both care and patience, because almost all of the more systematic means of physical training are tiresome, and excite none of the interest which belongs to the exercises of outdoor life. The difficulty vanishes in the presence of ample means. Said a gentleman to me yesterday, "Colorado in summer, and horseback in winter—these are what cured me of nervousness."

The mental attitudes of the nervous man demand of his physician the most careful attention, nor can we afford to disregard anything in his ways of life or his habits of thought and action. We must determine for him how far and how much he shall use his mind; whether or not it is well for him to continue his work, whatever it be, what his amusements should be. The careful student of such cases will find in the individuality of his cases the need for the most minute of such studies, and, above all, he will learn that the more fully he commands the confidence of his patient the more can he effect. Such people are greatly helped by a word or two of decisive promise or of reassurance, and since a part of the treatment of the acquired habit of nervousness must consist in mental and moral training, it becomes needful at times to make the patient comprehend that he himself has some control over his symptoms, or, at least, that constantly to yield to them is to insure defeat, and that constantly to struggle in a manly way is sure to bring nearer the day of perfect self conquest.

But, besides these obvious means, we have some agents, the careful employment of which is of essential value in general nervousness. First among them is the use of water in its varied forms of application, a too much neglected therapeutic aid. We have no water-cures in this country conducted on scientific principles, and by men of experience and intelligence, yet much may be done by the aid of the shower-bath, the douche, and the pack in the patient's own home. Alcohol would also have its use, and, indeed, has its use, but it is so immediate and perfect a relief to many people who are physically nervous, that I for one often fear to use it.

Then come the drugs. There is a form of cardiac and vasal irritability which is found in many nervous people, and which is most distressing. I have now a patient who never receives a guest, or even the best known visitor, without becoming pale, even white to faintness, and without having the most violent palpitations. Another never opens a letter without like symptoms. In such cases full doses of digitalis long kept up, are most valuable, and are in many cases capable of helping to form a habit of insensibility to such influences when once we have succeeded in bringing the nutritive system into perfect condition.

Digitalis has, I think, no power to aid the other symptoms of general nervousness. It helps the vasal and cardiac troubles, but not the tremor nor yet the mental symptoms, the indecision, the shyness, the timidity, the self-consciousness, as chloral does, at least for the time.

Of more general value are both opium and cannabis, but neither ought to be used very long, and when taking them the patient should not know what he is taking, and, above all, they should be used in very small doses, and perhaps I ought to say what I mean by small doses. In such cases I give of morphia 1-20 grain, with 1-20 of cannabis, and a varying amount of aloæ ex. or rhubarb, and if these doses are too strongly felt, I give even a less amount.

Among the general nerve tonics arsenic stands high, but the secret of its use lies in its long continuance. The bromides are also of the utmost utility. Among them I prefer the lithium bromide, which I introduced into medical use some years ago, and which has now stood the test of my own longer experience, as well as that of many French and German therapeutists, who have come to regard it as the most valuable of the bromides. It should be given in some simple bitter for many months, and in doses of not more than five or ten grains, thrice a day. I often use the bromide of lithium and the bromide of arsenic together in solution. The valerianates have also their uses, and I am fond of combining the three valerianates of quinia, zinc, and strychnia in the pill form. I am much more doubtful of the prolonged usefulness of such agents as assafœtida and sumbul, which, however, are often effective to tide over periods of increased nervousness.—*Med. News and Library.*

